# Palmitoleic Acid Inhibits Hepatotoxic Effects by Reducing Trimethylamine-*N*-Oxide (TMAO) Formation in High L-Carnitine-Treated Mice

**DOI:** 10.3390/nu16213599

**Published:** 2024-10-23

**Authors:** Qingzheng Han, Yu Liu, Xinyu Liu, Yue Geng, Qiu Wu, Hang Xiao

**Affiliations:** 1 College of Life Sciences, Shandong Normal University, Ji’nan 250358, China; yelulinn@gmail.com (Q.H.); liuyu157@163.com (Y.L.); liu054648@163.com (X.L.); gengy@sdnu.edu.cn (Y.G.); 2 Department of Food Science, University of Massachusetts, Amherst, MA 01003, USA; hangxiao@gmail.com

**Keywords:** POA, liver function, TMAO, antioxidant, microbiota

## Abstract

Background/Objectives: This study investigated the effects of palmitoleic acid (POA) consumption on liver function, intestinal microbiota, and trimethylamine-*N*-oxide (TMAO) levels in the serum of mice treated with 3% L-carnitine drinking water. The purpose was to highlight the impact of POA on liver injury associated with high L-carnitine intake. Methods: A correlation analysis was carried out. The physiological and biochemical results showed that the administration of POA could alleviate liver injury induced by high L-carnitine ingestion, as reflected by a reduction in liver function indices (ALT, AST, AKP, and TBA activities) and modulation of antioxidant enzyme activities (SOD, GSH-Px, MDA, and RAHFR). The study also monitored the levels of total cholesterol (TC), triglycerides (TG), low-density lipoprotein cholesterol (LDL-C), and high-density lipoprotein cholesterol (HDL-C). Additionally, to assess the impact of POA on intestinal microbiota, we conducted a 16S rRNA high-throughput sequencing analysis. Results: The findings indicated that POA administration resulted in lower levels of TMAO in treated mice. Furthermore, POA could regulate the composition of intestinal microbiota in L-carnitine mice, particularly affecting *Bacteroides vulgatus*, *Parabacteroides distasonis*, *Alistipes shahii*, *Lachnospiraceae* NK4A136 group, and *Parasutterella secunda*, which were closely related to liver injury. Conclusions: In summary, POA could repair liver damage caused by high intake of L-carnitine by regulating the distribution of intestinal flora and subsequently decreasing serum TMAO levels.

## 1. Introduction

The omega-3, -6, and -9 fatty acids in oils have been well documented, but ω-7 fatty acids (omega-7) have been poorly studied because of their low levels in common crops [1,2]. As the most representative ω-7 fatty acid, palmitoleic acid (POA) can only be found in abyssal fish (such as anchovies, sardine, bigeye tuna, etc.) and certain plant foods (macadamia nut oil, sea buckthorn fruit oil, seaweed, etc.) [3,4]. It is insoluble in water, but easily soluble in alkali solution and organic solvents such as ether, chloroform, hexane, and ethyl acetate. It is a colorless and transparent liquid at room temperature [3,4].

POA was found to be abundant in *Decaisnea fargesii* oil from Sichuan province of China [5]. The content of POA in *Decaisnea fargesii* oil was as high as 55.25–64%, which was significantly higher than that of salmon (6.0%), cod liver oil (7.1%), Queensland fruit oil (17%), Shanxi sea buckthorn fruit oil (13%), hairy dogwood seed oil (3.64%), and any other oils [6]. At the same time, several reports related to the advantages of POA have attracted attention [7,8]. Their results showed that POA could effectively antagonize some chronic sub-health problems, such as metabolic syndrome, glucose and lipid metabolism disorders, and inflammation [9,10,11]. Even though POA has a certain function in terms of obesity and diabetes, whether it can mitigate L-carnitine-induced liver injury has not been reported.

Liver injury induced by high ingestion of L-carnitine has been developing into a new kind of hepatotoxicity. Similar to the hepatotoxicity of food hazards such as benzopyrene and aflatoxin [12,13,14], the hepatotoxicity induced by high L-carnitine is also a toxic reaction worthy of attention. L-carnitine can be metabolized to trimethylamine (TMA) by gut microbiota and further converted into trimethylamine-*N*-oxide (TMAO) in the liver, leading to liver damage [15,16]. The liver damage induced by high L-carnitine ingestion is a very serious problem, and therefore, it is necessary to further develop safe and active ingredients that can prevent such damage.

Given this background, we hypothesized that POA can modulate the composition of the intestinal microbiota, thereby reducing liver damage caused by L-carnitine. We aimed to study the changes in liver function indicators and intestinal microbiota composition in mice after intervention with POA, and performed a correlation analysis to explore the potential mechanisms by which POA reduces serum levels of TMAO. This will help deepen our understanding of the protective effects of POA and provide a scientific basis for its clinical application.

## 2. Materials and Methods

### 2.1. Materials and Chemicals

POA and L-carnitine were bought from Innochem Co., Ltd. (Beijing, China). Assay kits for aspartate aminotransferase (AST), alanine aminotransferase (ALT), total protein (TP), total bile acid (TBA), alkaline phosphatase (AKP), albumin (ALB), malondialdehyde (MDA), superoxide dismutase (SOD), restraining ability to hydroxyl free radicals (RAHFR), glutathione peroxidase (GSH-PX), triglyceride (TG), total cholesterol (TC), low-density lipoprotein cholesterol (LDL-C), and high-density lipoprotein cholesterol (HDL-C) were purchased from Nanjing Jiancheng Bioengineering Institute (Nanjing, China). Deuterium hydrogen oxide (D_2_O) was bought from Kerunda Medical Equipment Co., Ltd. (Jinan, China).

### 2.2. Animal Experiment and Sample Collection

Healthy male Kunming mice (*n* = 30), 18~22 g weight, were purchased from Charles River Co., Ltd. (Beijing, China). All mice used in this study were males to eliminate the variability in metabolic responses that can occur due to hormonal differences between sexes. Our specific animal experimental design is outlined below.

Population: We selected 30 healthy male Kunming mice, weighing 18~22 g, as experimental animals. Intervention: After a one-week adaptation period, the mice were randomly divided into five groups of six each, with three groups designated as POA groups. Mice in these groups received different doses of POA (50, 100, and 200 mg/kg) and were given 3% L-carnitine water to drink. The choice of a 3% L-carnitine solution was based on our previously published studies, which demonstrated that this concentration could induce measurable changes in liver function without causing acute toxicity [15]. Comparison: The remaining two groups were designated as the normal (chow) group and the model group. Mice in the normal group did not receive POA or 3% L-carnitine but were given and drank normal saline, whereas the model group mice were given 3% L-carnitine and drank normal saline. Outcome: After eight weeks, the mice were euthanized. Serum, liver, urine, and fecal samples were collected and stored at −80° C. Liver function indicators, changes in intestinal microbiota composition, and serum levels of TMAO were measured to assess the hepatoprotective effects of POA as experimental results.

L-carnitine and POA were administered via oral gavage to ensure precision in dosing and control over the administration process. Before performing the gavage, our operators received relevant training to ensure the accuracy of the procedure and to minimize discomfort and risk of injury to the animals during administration. Animals were closely monitored throughout the gavage treatment, and any health issues or abnormal behaviors were addressed immediately. Our experiments were carried out according to the Guidelines for the Care and Use of Animals, and this study was evaluated and approved by the Ethics Committee of Shandong Normal University (Approved No. AEECSDNU2022014), and the experimental procedure was carried out according to the Guideline of Experimental Animal Administration published by the State Committee of Science and Technology of the People’s Republic of China.

### 2.3. Determination of Serum Clinical Indexes and Hepatic Biomedical Indexes

The levels of ALT and AST in serum were tested by using ALT and AST kits. The levels of AKP, TP, ALB, TBA, SOD, RAHFR, MDA, GSH-PX, LDL-C, HDL-C, TC, and TG in liver were also detected by using kits. Liver homogenate was obtained by using the following methods: 4.5 mL normal saline was added to liver tissue (0.5 g), which was then homogenized using a tissue lyser. The mixture were centrifuged at 3500 rpm for 10 min, and the supernatant was stored at −80 °C.

### 2.4. Histopathological Observation of Liver Tissues

Tissues (0.25 m^2^) used for histopathological observation were cut from the left lobe of the liver. The tissues were fixed in 4% paraformaldehyde and then were embedded in paraffin and cut into 6 μm sections for hematoxylin and eosin (H&E) dye staining.

### 2.5. Intestinal Microflora Analysis

DNA extraction, PCR amplification, creation of sequencing libraries and evaluation of sequences were carried out by using the method of Zhu et al. with some modifications [17]. The reads were merged using FLASH (V1.2.7), and high-quality sequencing reads were screened with QIIME (V1.7.0) [18,19]. The screened reads were then selected by comparing with the Gold Database [20], and sequences with 97% similarity were distributed to an OTU (Operational Taxonomic Unit). Rarefaction analysis, Venn diagrams, Good’s coverage, and alpha diversity analyses were carried out using QIIME (V1.7.0). Chao1 and Ace indexes were used to describe the richness of flora, and Shannon and Simpson indices were used to describe the community diversity [18].

Venn graphs and PCA (Principal Component Analysis) graphs were evaluated using the Ggplot2 package in R (Version 4.2.3). UPGMA (Unweighted Pair Group Method with Arithmetic Mean) clustering was carried out by using QIIME (V1.7.0). The abundance of microorganisms at the phylum level was tested based on the top 7 dominant genera. LEfSe (Linear Discriminant Analysis Effect Size), heatmap, and Spearman’s nonparametric correlation analyses were conducted in R [21,22].

### 2.6. Measurement of TMAO in Serum

In our previous research, we established a reliable, simple, and effective method for the determination of TMAO in mouse serum and urine using an Agilent 6460C QQQ LC-MS/MS. Serum samples were prepared through preprocessing, followed by LC-MS/MS analysis under optimized conditions, and the concentrations of TMAO in the serum and urine samples were calculated based on standard curves [14].

### 2.7. Correlation Analysis and Statistical Analysis

In order to more closely examine the connection between gut microbiota and indices related to liver injury, nonparametric Spearman’s rho correlation analyses were conducted utilizing R (version 4.2.3). Before conducting parametric statistical analyses, we first performed normality tests on all major variables. Specifically, the Shapiro–Wilk test was used to assess the normal distribution of the data, ensuring the appropriateness and validity of subsequent parametric analyses. Data are presented as mean ± standard deviation (SD). Statistical analysis, specifically ANOVA tests, was performed using SPSS 20, with a *p*-value of less than 0.05 considered to indicate statistical significance.

## 3. Results

### 3.1. Effect of POA on Body Weight, Liver Weight, and Hepatosomatic Index (HI) of Mice Treated with High L-Carnitine

Table 1 shows the impact of POA on body weight, liver weight, and HI in mice subjected to high L-carnitine diets. These findings suggest that all measured parameters were elevated in the carnitine group compared to the control. However, supplementation with POA at varying doses appears to mitigate these effects, particularly at the highest tested dose of 200 mg/kg, where a significant reduction in the hepatosomatic index was observed (*p* < 0.05). This implies that POA may have a protective effect against weight gain and liver enlargement induced by high L-carnitine consumption.

### 3.2. Effects of POA on Serum Transaminase, Liver Function, Lipid Metabolism, and Antioxidant Capacity in High L-Carnitine Mice

**Figure 1:**
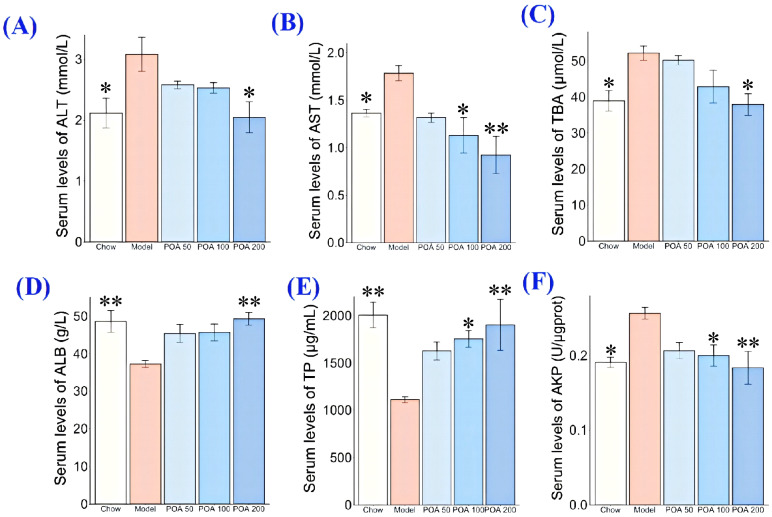
Effects of POA on the transaminase levels and the four indicators of liver function in livers of high L-carnitine-treated mice—ALT (**A**), AST (**B**), TBA (**C**), ALB (**D**), TP (**E**), and AKP (**F**). (Asterisks (*) indicate levels of statistical significance where * represents *p* < 0.05 and ** represents *p* < 0.01 compared to the control group).

In mice with high levels of carnitine, AST and ALT serum levels increased from 1.36 ± 0.09 mmol/L and 2.12 ± 0.6 mmol/L in the normal group to 1.79 ± 0.19 mmol/L and 3.08 ± 0.68 mmol/L, respectively (*p* < 0.05). However, these levels decreased in mice treated with POA at 50, 100, and 200 mg/kg, with AST dropping by 16.2%, 17.9%, and 33.6% respectively (*p* > 0.05, *p* > 0.05, *p* < 0.05) as seen in Figure 1B. Similarly, ALT levels in the POA groups showed a comparable decrease compared to the carnitine group (Figure 1A).

AKP, TBA, TP, and ALB are indicators of liver function and help assess liver damage [23]. In Figure 1C,F, it is shown that in mice fed with L-carnitine, the levels of AKP and TBA in the liver significantly increased (*p* < 0.05), while TP and ALB levels significantly decreased (*p* < 0.01), indicating that L-carnitine intake might cause liver injury. When these mice were treated with POA at doses of 50, 100, and 200 mg/kg, the AKP levels decreased by 19.5%, 22.0%, and 28.4% (*p* > 0.05, *p* < 0.05, *p* < 0.01) (Figure 1F); TBA levels decreased by 3.7%, 18.3%, and 27.3% (*p* > 0.05, *p* > 0.05, *p* < 0.05) (Figure 1C); TP levels increased by 46.3%, 57.9%, and 71.2% (*p* > 0.05, *p* < 0.05, *p* < 0.01) (Figure 1E); and ALB levels increased by 21.7%, 22.5%, and 32.2% (*p* > 0.05, *p* > 0.05, *p* < 0.01) (Figure 1D). These results indicate that POA has the potential to improve liver function in L-carnitine mice.

**Figure 2:**
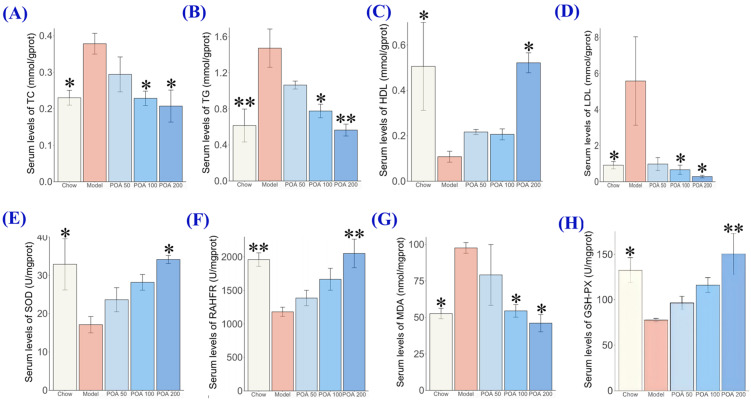
Effects of POA on the antioxidant capacity and lipid homeostasis of liver in high L-carnitine-treated mice—TC (**A**), TG (**B**), HDL-C (**C**), LDL-C (**D**), SOD (**E**), RAHFR (**F**), MDA (**G**) and GSH-PX (**H**). (Asterisks (*) indicate levels of statistical significance where * represents *p* < 0.05 and ** represents *p* < 0.01 compared to the control group).

Liver damage is often accompanied by lipid metabolism disorders [24]. As shown in Figure 2A,D, in the model group, the levels of TC and TG in the liver increased by 64.3% and 139.4%, respectively (*p <* 0.05, *p* < 0.01), and HDL-C and LDL-C also showed abnormal conditions. After treatment with 50, 100, and 200 mg/kg of POA, TC levels decreased by 22.2%, 39.6%, and 45.2%, respectively (*p* > 0.05, *p* < 0.05, *p* < 0.05) (Figure 2A), and TG levels decreased by 27.8%, 47.3%, and 61.7% (*p* > 0.05, *p* < 0.05, *p* < 0.01) (Figure 2B). Compared with the high L-carnitine model group, HDL-C levels significantly increased (Figure 2C), while LDL-C levels decreased (Figure 2D). These results indicate that POA may alleviate liver damage in mice induced by high L-carnitine intake through modulation of lipid metabolism.

Oxidative stress is closely linked to lipid metabolism [25], hence the activities of SOD, RAHFR, MDA, and GSH-Px in liver tissue were measured, with results displayed in Figure 2E,H. The MDA in the model group (97.59 ± 8.98 U/mgprot) was significantly higher than that of the normal group (52.59 ± 8.77 U/mgprot) (*p* < 0.05), and the GSH-PX level (77.58 ± 4.11 nmol/mgprot) was significantly lower than that of the normal group (132.46 nmol/mgprot) (*p* < 0.05). Additionally, after treatment with 50, 100, and 200 mg/kg of POA, the MDA levels decreased by 19.0%, 44.2%, and 52.8%, respectively (*p* > 0.05, *p* < 0.05, *p* < 0.05) (Figure 2G), but the GSH-PX levels increased by 24.4%, 49.6%, and 93.6%, respectively (*p* > 0.05, *p* > 0.05, *p* < 0.01) (Figure 2H). Compared to the normal group, the SOD and RAHFR levels in the carnitine group decreased by 47.9% and 22.9%, respectively (*p* < 0.05, *p* < 0.01), while SOD and RAHFR significantly increased after treatment with 50, 100, and 200 mg/kg of POA. Together, these results indicate that POA may alleviate liver damage in mice induced by high L-carnitine intake by modulating lipid metabolism pathways associated with oxidative stress.

### 3.3. Effect of POA on the Histopathology of Liver Tissues and the TMAO Content in Serum of Mice with High Carnitine Levels

**Figure 3:**
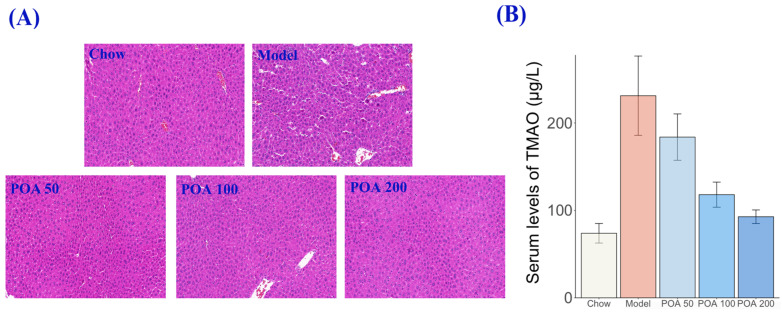
Effects of POA on histopathological changes in the livers of mice treated with high doses of L-carnitine—liver hepatocytes stained with H&E (Normal, Model, POA 50, POA 100, and POA 200) (**A**), and the TMAO content in the serum (**B**).

To further confirm the counteracting effect of POA against liver toxicity in mice with high L-carnitine, liver tissues were subjected to H&E staining, with the findings presented in Figure 3A. The liver cells in normal mice exhibited a normal structure, including distinct nuclear membranes, clear nucleoli, and no signs of necrosis. In contrast, the liver cells in mice with high carnitine showed marked necrosis, cytoplasmic vacuolization, and blurred cellular boundaries. However, post-treatment with palmitic acid led to an improvement in the liver cell slices, with a reduction in cytoplasmic vacuoles and progressively clearer cell boundaries. Additionally, after treatment with POA, the significantly elevated levels of TMAO in the serum of mice with high carnitine intake, as compared to the normal group, also decreased (Figure 3B). These results suggest that POA may mitigate hepatotoxicity induced by high L-carnitine.

### 3.4. Effect of Palmitic Acid on Gut Microbiota of High L-Carnitine-Treated Mice

**Figure 4:**
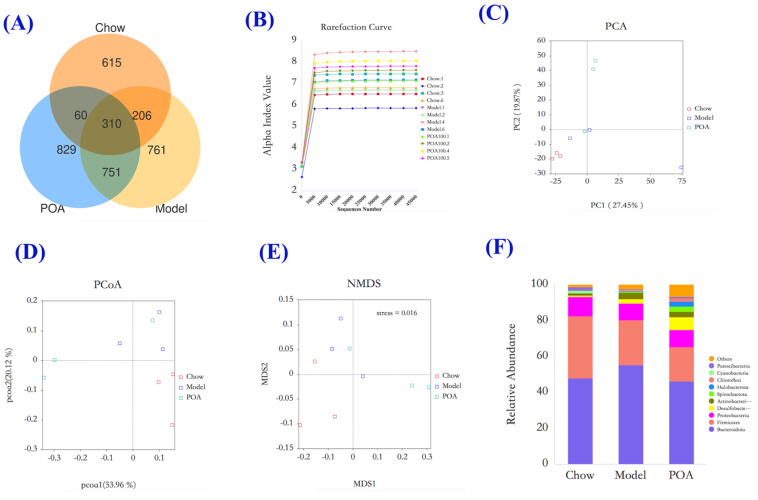
Venn diagram (**A**) of the gut microbiota of mice in different groups, rarefaction curve analysis (**B**), PCA plot (**C**), PCoA plot (**D**), NMDS clustering plot (**E**), and bar chart of relative species abundance at the phylum level (**F**).

To further elucidate the mechanisms by which POA alleviates liver damage induced by high L-carnitine in mice, we also examined the distribution of gut microbiota in mice treated with 200 mg/kg POA for sampling. In microbial diversity analysis, it is necessary to verify the sufficiency of the sequencing data volume to accurately reflect the species diversity in the sample. The dilution curve used to test this indicator indirectly reflects the richness of species in the sample. When the curve becomes flat or reaches a plateau, it can be considered that the sequencing depth has basically covered all the species in the sample. According to our dilution curve analysis results, the sequencing depth covered rare new phylotypes and most of the diversity (Figure 4B).

The Venn diagram of fecal analysis results showed that the Chow, Model, and POA groups of mice shared 310 OTUs. The Chow and Model groups had 206 OTUs in common; the Model and POA groups shared 751 OTUs; and the Chow and POA groups had 60 OTUs in common. There were 615 unique OTUs in the feces of the Chow group, 761 unique OTUs in the Model group, and 829 unique OTUs in the POA group (Figure 4A).

Principal component analysis (PCA), principal coordinates analysis (PCoA), and non-metric multidimensional scaling (NMDS) are all dimensionality-reduction ordination methods, which are techniques used to simplify datasets [26,27]. The PCA results showed that the two main components, PC1 and PC2, contributed 27.45% and 19.87%, respectively (Figure 4C). The PCoA results indicated that the two main components (pcoa1 and pcoa2) contributed 53.96% and 20.12%, respectively (Figure 4D). The NMDS analysis results showed a stress value of 0.016, indicating that the higher the similarity between samples, the more clustered they are in the center. According to Figure 4C,E, the Chow, Model, and POA groups demonstrate a good separation trend.

**Figure 5:**
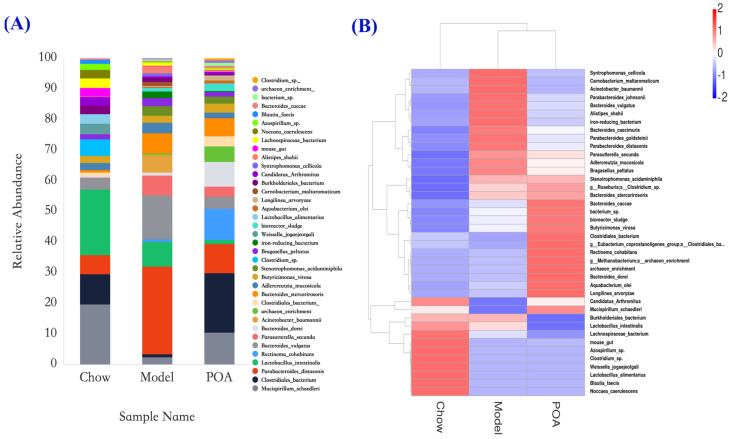
Relative abundance of gut microbiota at the genus level in mice of different groups (**A**) and an abundance heatmap at the genus level (**B**).

To further investigate the effects of POA on the gut microbiota of mice with high levels of carnitine, we conducted a detailed analysis of the microbial composition at different taxonomic levels. At higher taxonomic levels, compared to the control group, the induction of high carnitine levels significantly increased the abundance of *Bacteroidota*, *Actinobacteria*, and *Desulfobacterota*. After the addition of POA, the abundances of *Bacteroidota*, *Actinobacteria*, and *Desulfobacterota* were downregulated (Figure 4F). At the genus level, compared to the control group, the abundance of species such as *Parabacteroides distasonis*, *Bacteroides vulgatus*, and *Parasutterella secunda* in the intestines of mice in the high-carnitine group increased, while the abundance of *Mucispirillum schaedleri* and *Clostridiales* bacterium decreased. After POA intervention, the abundances of *Parabacteroides distasonis*, *Bacteroides vulgatus*, *Parasutterella secunda*, and other species were reduced, while the abundances of *Mucispirillum schaedleri*, *Clostridiales* bacterium, and other genera increased (Figure 5A,B).

**Figure 6:**
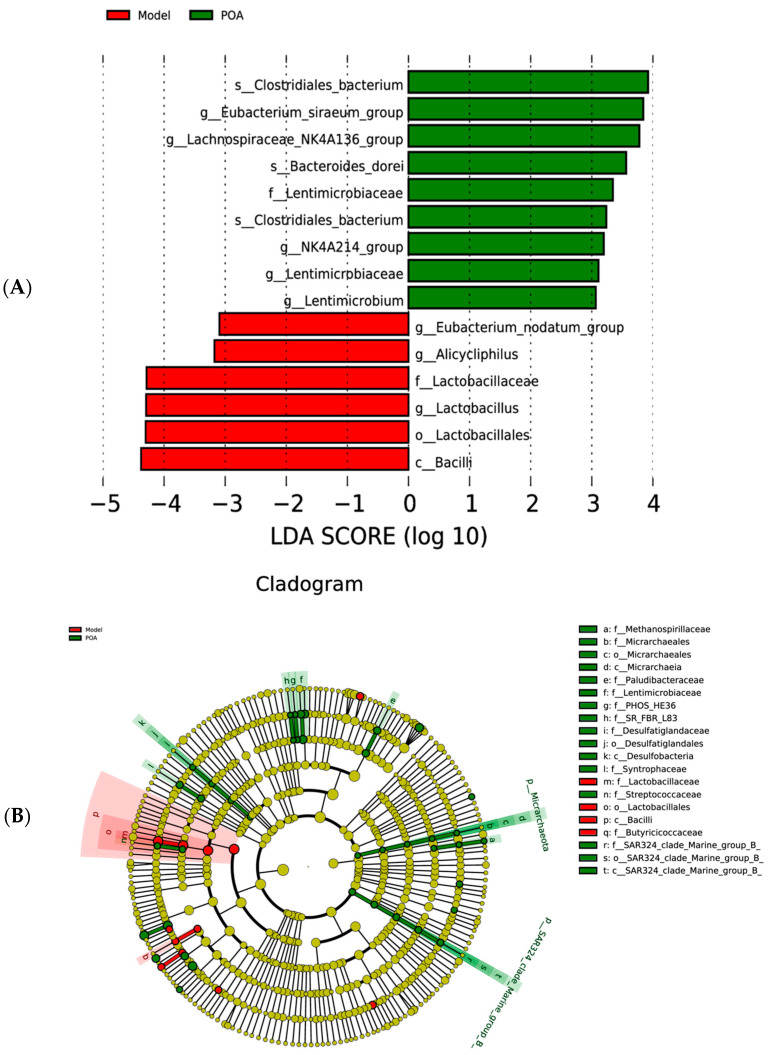
LEfSe analysis of gut microbiota in the Model group and in POA group mice—Significant difference chart (**A**) and representative cladogram (**B**).

Before beginning the significance analysis, we conducted a LEfSe analysis using a linear discriminant analysis (LDA) score threshold of log10 > 3.5, resulting in an evolutionary branching diagram and a significance difference chart. Compared with the model group of mice treated with high L-carnitine, in the feces of the POA-treated group of mice, at the species level, the content of *Bacteroides dorei* and *Clostridiales* bacterium significantly increased; at the genus level, the content of the *Eubacterium siraeum* group and the *Lachnospiraceae* NK4A136 group significantly increased, while the content of *Lactobacillus* was significantly downregulated; at the family level, the content of *Lactobacillaceae* was significantly downregulated; at the order level, *Lactobacillales* was significantly downregulated; and at the class level, the content of *Bacilli* was significantly downregulated (Figure 6A,B).

### 3.5. Correlation Analysis of the Liver Injury Index and Microbiota

To determine the potential association between the gut microbiota and liver damage, we conducted a Spearman correlation analysis, taking into account the results of the LEfSe analysis and the hierarchical classification of phylum, class, order, family, genus, and species within the microbiota. This analysis focused on eight bacterial species (*Lactobacillus, Bacteroides vulgatus, Parabacteroides distasonis, Alistipes shahii, Lachnospiraceae* NK4A136 group, *Eubacterium siraeum* group, *Mucispirillum schaedleri*, and *Parasutterella secunda*) and their relationships with liver damage-related indicators (TMAO, ALT, AST, ALB, TBA, TP, AKP, TG, LDL-C, HDL-C, TC, RAHFR, SOD, MDA, and GSH-Px).

**Figure 7:**
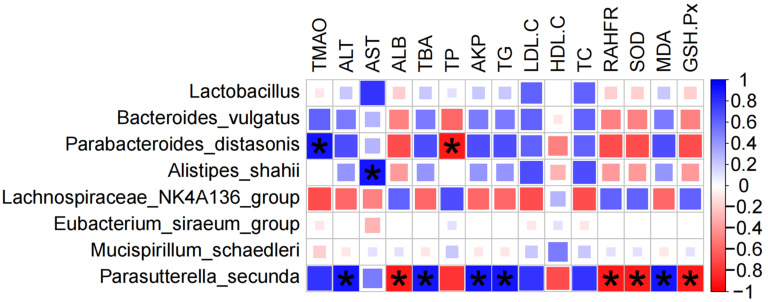
Spearman correlation heatmap of gut microbiota composition and liver injury markers. (Asterisks (*) indicate statistically significant correlations (*p* < 0.05)).

The results showed that the liver damage indicators had significant associations with *Bacteroides vulgatus*, *Parabacteroides distasonis*, *Alistipes shahii*, *Lachnospiraceae* NK4A136 group, and *Parasutterella secunda* (Figure 7). Specifically, the *Lachnospiraceae* NK4A136 group showed positive correlations with TMAO, ALT, AST, TBA, AKP, TG, LDL, TC, and MDA, and negative correlations with ALB, TP, SOD, and GSH-Px, suggesting that it may be an important bacterial strain inducing liver damage. Conversely, *Bacteroides vulgatus*, *Parabacteroides distasonis*, and *Parasutterella secunda* showed positive correlations with ALB, TP, SOD, and GSH-Px (*p* < 0.05), and negative correlations with TMAO, ALT, AST, TBA, AKP, TG, LDL, TC, and MDA (*p* < 0.05). Similarly, *Alistipes shahii* also demonstrated a significant negative correlation with AST, indicating that *Bacteroides vulgatus*, *Parabacteroides distasonis*, *Alistipes shahii*, and *Parasutterella secunda* may be important bacterial strains for preventing liver damage.

## 4. Discussion

In this study, mice that had ingested 3% L-carnitine water were used as the model group to discuss the effects of POA on the gut microbiota, liver function, and metabolite distribution in mice treated with high L-carnitine. The study aimed to explore how POA modulates the distribution of gut microbiota to antagonize liver toxicity induced by high L-carnitine. The mechanism and principles of POA’s hepatoprotective effects were revealed through the measurement of transaminases in mouse serum, antioxidant capacity, lipid metabolism levels in liver tissues, and changes in the distribution of gut microbiota.

During the feeding period, there was no significant difference in the average intake of food and water among the groups. Compared to normal mice, those fed high doses of L-carnitine exhibited significantly increased body weight, liver weight, and liver-to-body weight ratios, consistent with our previous research findings [28]. Mice fed with POA showed a noticeable decline in body weight, particularly at higher doses, a trend also observed by Souza et al. [29], who investigated the role of POA in alleviating immune-metabolic disturbances caused by a high-fat diet. Furthermore, the liver injury indices (AST and ALT levels) were significantly higher in mice treated with high doses of L-carnitine compared to the normal group, but these markers significantly decreased in the serum of mice after POA treatment. This result is in line with other studies demonstrating the hepatoprotective effects of POA [30], further supporting the potential anti-oxidative and anti-inflammatory mechanisms of POA in liver cells.

Significant changes were observed in the liver tissue content of ALB, TP, TBP, and AKP, which are related to liver function [31]. The trends in the high L-carnitine group indicated liver function damage compared to the normal group, whereas POA treatment could alleviate the liver toxicity induced by high L-carnitine. In terms of antioxidant capacity in liver tissues, the MDA content was significantly higher in the high L-carnitine group compared to the normal group, but it decreased in a dose-dependent manner under the influence of POA, suggesting high L-carnitine-induced oxidative stress in mouse liver and increasing MDA levels, while POA could mitigate this oxidative stress, significantly reducing the elevated MDA levels. This effect was more pronounced at lower doses of POA, while medium and high doses had similar effects that were less distinct than the low dose. Additionally, similar to MDA, the contents of RAHFR and GSH-Px were significantly higher in the liver tissues of the high L-carnitine group compared to the normal group, but their levels significantly decreased after treatment with different doses of POA, indicating that POA can alleviate oxidative stress caused by high L-carnitine and enhance the antioxidant capacity of mice. The SOD content in the liver tissues of the high L-carnitine group was significantly lower than in the normal group, but it gradually increased after treatment with different doses of POA and exceeded the levels in the normal group. This is consistent with our previous studies on the dose-dependent alleviation of high L-carnitine-induced liver damage by quercetin, [28] suggesting that POA also has a similar effect to quercetin, alleviating oxidative stress induced by high doses of L-carnitine and enhancing the antioxidant capacity of mice.

These results indicate that POA could downregulate oxidative damage in mice treated with high L-carnitine and effectively enhance their antioxidant capacity. Furthermore, studies have shown that liver lipid metabolism is associated with the antioxidant defense system [32]. Consuming high L-carnitine in mice induced an upregulation of TC, TG, and LDL-C, and a downregulation of HDL-C, but after intervention with POA, the TC levels showed a downward trend compared to the high L-carnitine model group, TG levels also showed a downward trend, while HDL-C levels increased compared to the model group, and LDL-C levels showed a downward trend and gradually stabilized, indicating that POA could mitigate the impact of high L-carnitine on liver lipid metabolism levels and regulate the lipid metabolism homeostasis in the liver of high L-carnitine-treated mice.

A substantial amount of animal experimental research has shown that the gut microbiota is closely related to human health [33]. The gut microbiota residing in human intestines participates in various vital activities of the body, and disruption of its compositional structure can induce the occurrence of multiple diseases. Dysbiosis of the gut microbiota has become a pathogenic mechanism for many diseases [33,34]. It has been proven that the gut microbiota participates in the metabolism of L-carnitine in mice, and prolonged intake of high levels of L-carnitine can induce dysbiosis in the gut microbiota of mice [28,35]. Simultaneous analysis based on 16S rRNA high-throughput sequencing revealed that long-term consumption of high L-carnitine causes dysbiosis of the gut microbiota, while POA can mitigate the impact of high L-carnitine intake on the distribution of gut microbiota in mice. Rarefaction curves indicated that the sequencing data were sufficient and reliable. Clustering analysis through Venn diagrams, PCA, PCoA, and NMDS plots showed a clear separation trend between the normal group and POA-treated mice compared to the model group. Further compositional analysis of gut microbiota at different taxonomic levels, revealed through bar graphs of relative abundances and heat map and LEfSe analyses, showed that in the model group, the abundances of *Lactobacillus*, as well as *Parabacteroides distasonis* and *Bacteroides vulgatus* from the phylum *Bacteroidota*, were significantly increased compared to the normal group. These abundances were reduced toward normal levels following treatment with POA. Conversely, the abundance of *Clostridiales bacterium*, *Eubacterium siraeum* group, and *Lachnospiraceae* NK4A136 group initially decreased compared to the normal group but increased again after treatment with POA.

Existing studies indicate that *Parabacteroides distasonis, Bacteroides vulgatus, Lachnospiraceae* NK4A136 group, and *Alistipes shahii* are all closely related to liver injury. Specifically, *Parabacteroides distasonis* can increase intestinal bile salt hydrolase activity and inhibit the intestinal FXR signaling pathway, reducing TCDCA levels in the liver. This reduction in TCDCA helps to inhibit hepatocyte MPT-Caspase-11 pyroptosis and stellate cell activation, thereby improving liver fibrosis [36]. *Bacteroides vulgatus* can improve atherosclerosis by lowering blood endotoxin levels and produces N-acetylglucosamine to promote the growth of *Akkermansia*, thereby reducing obesity and regulating glucose metabolism [37,38]. *Lachnospiraceae* NK4A136 group has been identified as a key biomarker for liver toxicity reactions caused by polylactic acid microplastics in recent studies [39]. *Alistipes shahii* plays a protective role in the fecal microbiota of patients with decompensated cirrhosis and acute hepatic encephalopathy; a reduction in its abundance is associated with an increased recurrence of hepatic encephalopathy. Therefore, a decrease in *Alistipes shahii* is associated with the progression of cirrhosis to a decompensated state [40,41].

To further investigate the potential association between the composition of the gut microbiota and liver damage, this study conducted another Spearman correlation analysis. The results are shown in Figure 7. The *Lachnospiraceae* NK4A136 group exhibited positive correlations with TMAO, ALT, AST, TBA, AKP, TG, LDL, TC, and MDA, while showing negative correlations with ALB, TP, SOD, and GSH-Px. This suggests that it might play a crucial role in promoting liver damage. In contrast, *Bacteroides vulgatus, Parabacteroides distasonis*, and *Parasutterella secunda* were positively correlated with ALB, TP, SOD, and GSH-Px (*p* < 0.05), and negatively correlated with TMAO, ALT, AST, TBA, AKP, TG, LDL, TC, and MDA (*p* < 0.05). *Alistipes shahii* also displayed a significant negative correlation with AST, which supports the idea that *Bacteroides vulgatus, Parabacteroides distasonis, Alistipes hahii*, and *Parasutterella secunda* may be important bacterial strains for preventing liver damage. The results of the Spearman correlation analysis are consistent with the literature reports stating that the *Lachnospiraceae* NK4A136 group is closely related to liver damage and that *Bacteroides vulgatus, Parabacteroides distasonis,* and *Alistipes shahii* have a protective effect on the liver [36,37,38,39,40,41]. However, there is limited research on *Parasutterella secunda*, and strong evidence linking it to liver damage has not yet been found. Nonetheless, in this study, its correlation with changes in liver indicators is particularly significant, suggesting that it may also play a substantial role in protecting the liver and reducing damage.

Finally, although this study has revealed some intestinal microbiota characteristics associated with the treatment of L-carnitine-induced liver injury using POA, the reconstitution of the microbiota has not yet been validated. Moving forward, representative microbiota identified in the experiment, especially the underreported *Parasutterella secunda*, could be selected for in vitro fermentation, followed by microbiota replenishment trials, to facilitate bidirectional validation at both in vitro and in vivo levels. This will enhance the whole animal-level experiment and supplement the logical process, making this study more objective and rigorous.

## 5. Conclusions

In summary, this experiment revealed the impact of moderate POA intervention on the distribution of gut microbiota in mice with liver injury induced by high L-carnitine intake. We performed a correlation analysis with liver injury assessment indicators, identifying some significant characteristic bacteria to explore some mechanisms by which POA may repair liver damage caused by high L-carnitine intake. This provides a promising direction for exploring new avenues to treat liver damage caused by high L-carnitine intake, with the hope of making an impact in clinical applications.

## Figures and Tables

**Table 1:** Body weight, liver weight, and hepatosomatic index (HI) of mice at the end of Week 8.

Determination Index	Group				
	Chow	Carnitine	POA 50	POA 100	POA 200
Initial Body Weight (g)	32.4	32.2	32.0	31.7	31.1
Final Body Weight (g)	47.75 ± 3.67	48.25 ± 0.94	47.58 ± 3.58	44.92 ± 4.87	44.40 ± 2.78
Liver Weight (g)	1.74 ± 0.136	1.90 ± 0.203	1.67 ± 0.429	1.62 ± 0.204	1.50 ± 0.099
Hepatosomatic Index (%)	3.68 ± 0.005	3.91 ± 0.004	3.53 ± 0.002	3.61 ± 0.002	3.38 ± 0.001

Hepatosomatic index = liver wt (g)/final body wt (g) × 100%. Data are expressed as the mean ± SD (6 mice in each group).

## Data Availability

The original contributions presented in the study are included in the article, further inquiries can be directed to the corresponding author.

## Abbreviations

POA	Palmitoleic acid
TMAO	Trimethylamine *N*-oxide
IL-1	Interleukin-1
IL-6	Interleukin-6
AST	Aspartate aminotransferase
ALT	Alanine aminotransferase
AKP	Alkaline phosphatase
TP	Total protein
ALB	Albumin
TBA	Total bile acid
TC	Total cholesterol
TG	Triglyceride
HDL-C	High-density lipoprotein cholesterol
LDL-C	Low-density lipoprotein cholesterol
SOD	Superoxide dismutase
GSH-Px	Glutathione peroxidase
MDA	Malondialdehyde
RAHFR	Restraining ability to hydroxyl free radicals
H&E	Hematoxylin and eosin
PCA	Principal component analysis
PLS-DA	Partial least squares–discriminate analysis
OPLS-DA	Orthogonal partial least squares–discriminant analysis
